# *Morinda citrifolia* and Its Active Principle Scopoletin Mitigate Protein Aggregation and Neuronal Apoptosis through Augmenting the DJ-1/Nrf2/ARE Signaling Pathway

**DOI:** 10.1155/2019/2761041

**Published:** 2019-05-02

**Authors:** Kishore Kumar S. Narasimhan, Deepthy Jayakumar, Prema Velusamy, Ashokkumar Srinivasan, Thangarajeswari Mohan, Divya Bhavani Ravi, Saraswathi Uthamaraman, Yogesh Kanna Sathyamoorthy, Namakkal Soorappan Rajasekaran, Kalaiselvi Periandavan

**Affiliations:** ^1^Department of Medical Biochemistry, Dr. ALM Post Graduate Institute for Basic Medical Sciences, University of Madras, Chennai, India; ^2^Cardiac Aging & Redox Signaling Laboratory, Division of Molecular & Cellular Pathology, Department of Pathology, University of Alabama, Birmingham, AL 35294, USA; ^3^Department of Anatomy, Dr. ALM Post Graduate Institute for Basic Medical Sciences, University of Madras, Chennai, India

## Abstract

Given the role of oxidative stress in PD pathogenesis and off-target side effects of currently available drugs, several natural phytochemicals seem to be promising in the management of PD. Here, we tested the hypothesis that scopoletin, an active principle obtained from *Morinda citrifolia* (MC), efficiently quenches oxidative stress through DJ-1/Nrf2 signaling and ameliorates rotenone-induced PD. Despite reducing oxidative stress, the administration of MC extract (MCE) has lessened protein aggregation as evident from decreased levels of nitrotyrosine and *α*-synuclein. *In vitro* studies revealed that scopoletin lessened rotenone-induced apoptosis in SH-SY5Y cells through preventing oxidative injury. Particularly, scopoletin markedly upregulated DJ-1, which then promoted the nuclear translocation of Nrf2 and transactivation of antioxidant genes. Furthermore, we found that scopoletin prevents the nuclear exportation of Nrf2 by reducing the levels of Keap1 and thereby enhancing the neuronal defense system. Overall, our findings suggest that scopoletin acts through DJ-1-mediated Nrf2 signaling to protect the brain from rotenone-induced oxidative stress and PD. Thus, we postulate that scopoletin could be a potential drug to treat PD.

## 1. Introduction

Although the causal mechanisms of Parkinson's disease (PD) remain elusive, excess production of reactive oxygen species (ROS), mitochondrial dysfunction, neuroinflammation, and environmental toxins are reported to promote the loss of dopaminergic neurons in PD [1]. Oxidative stress has been shown to induce misfolding, aggregation, and accumulation of such aggregates leading to the pathogenesis of many neurodegenerative diseases including PD [2]. Intracellular inclusions known as Lewy bodies (LBs) are regarded as a hallmark of common pathological manifestation in both familial and sporadic PD patients with *α*-synuclein (*α*-Syn) serving as the main component of LB [3]. *α*-Syn is natively unfolded and is prone to form fibrils during oxidative stress [4], indicating that redox signaling may play a significant role in the aggregation of *α*-Syn.

Previous studies have reported that the loss of antioxidant defense aggravates PD progression [5, 6]. A key example includes DJ-1/PARK7, a molecular chaperone known to regulate Keap1-Nrf2 signaling, which is the primary sensor for reactive electrophiles activating Nrf2 nuclear translocation and transactivation of the antioxidant response element (ARE) in a battery of cytoprotective genes facilitating protection from oxidative stress pathogenesis [7] including experimental models of PD [8]. Thus, pharmacological activation of the Nrf2 in the brain is likely to preserve neuronal health. Therefore, exploration for therapeutic compounds with lesser neurotoxic effects that activates Nrf2 signaling would be promising to treat PD.

In this context, identifying potential principles from medicinal plants would be ideal as plant extracts have been reported to have several therapeutic benefits, due to the synergistic effect of various natural ingredients [9]. However, such plant sources have not been in clinical practice or in global market due to the lack of scientific validation. *Morinda citrifolia* fruit extract (MCE) has potential antioxidant properties due to the presence of several active constituents (including scopoletin and quercetin) and protects skeletal muscle from apoptosis [10] and also prevents striatal degeneration [11] in experimental Parkinsonian rats. Here, we hypothesized that MCE and scopoletin prevent rotenone-induced oxidative stress and apoptosis through the activation of DJ-1/Nrf2 signaling and investigated its neuroprotective effects using *in vivo* (Sprague-Dawley rats) and *in vitro* (SH-SY5Y cells) models of PD.

## 2. Materials and Methods

### 2.1. Animals, Intranigral Rotenone Infusion, and Treatment

Adult male Sprague-Dawley rats were used in this study. All experiments were performed in accordance with the guidelines approved by the Institutional Animal Ethical Committee *(IAEC No. 01/09/12)*. Rats were divided into five groups (*n* = 10/gp). Group I served as control, while groups II to V were subjected to stereotaxic surgery. Group II served as sham controls and groups III-V were stereotaxically infused with rotenone to induce Parkinsonism. Briefly, rats were anaesthetized with ketamine hydrochloride and xylazine (80 mg/kg and 10 mg/kg; i.p.) and placed on a small animal stereotaxic frame (Stoelting, IL, USA). Rotenone dissolved in DMSO (1 *μ*g/1 *μ*l) was infused into the right ventral tegmental area (VTA, anterior-posterior (AP): 5.0 mm; laterally (L): 1.0 mm; dorso-ventral (DV): 7.8 mm) and into the right substantia nigra pars compacta (SNPc, AP: 5.0 mm; L: 2.0 mm; DV: 8.0 mm) at a flow rate of 0.2 *μ*l/min using a Hamilton 26-gauge needle [10, 11]. The infusion needle was left in place for additional five minutes for complete diffusion of the drug. Sham controls were infused with DMSO and polyethylene glycol in the ratio of 1 : 1 during stereotaxic surgery. Two weeks postsurgery, rats in groups IV and V were treated with levodopa (LD, 100 mg/kg with 25 mg/kg benserazide [12]) and ethyl acetate extract of *Morinda citrifolia* fruit (MCE, 150 mg/kg body weight), respectively, for the next 30 days. To determine the efficiency of intranigral infusion of rotenone, animals were subjected to behavioural analysis [11].

### 2.2. *In Vitro* Studies Using SH-SY5Y Cells

SH-SY5Y cells were initially grown in 1 : 1 mixture of DMEM and F12K Medium supplemented with 10% fetal bovine serum (*v*/*v*), 100 U/ml penicillin, and 100 *μ*g/ml streptomycin in a 25 cm^2^ and 75 cm^2^ vented culture flasks. The cultures were incubated at 37°C in 5% CO_2_/95% humidified air. When cells had reached 80–90% confluence in the flask, they were trypsinized and seeded onto 96-well plates or 6-well plates. Rotenone and scopoletin were dissolved in dimethyl sulfoxide (DMSO, final concentration of DMSO was 0.01%). Rotenone (500 nM) was used for 24 h to induce cell damage [13]. Preliminary studies were carried out with different concentrations (1 *μ*M, 10 *μ*M, 30 *μ*M, 50 *μ*M, and 100 *μ*M) and different time intervals (0 h, 1 h, 3 h, and 6 h) of scopoletin to fix the time of exposure and dosage using MTT assay and the optimal concentration was found to be 30 *μ*M pretreated for 3 h (Supplementary Figure 2). To investigate whether scopoletin protects cells from rotenone-induced cell death, cells were divided into three groups: control group, rotenone group (treated with rotenone for 24 h), and scopoletin group (pretreated with 30 *μ*M scopoletin for 3 h followed by exposure to rotenone).

### 2.3. Analysis of Apoptotic Cells by Flow Cytometry

The different stages of apoptosis in control and treated SH-SY5Y cells were analyzed by flow cytometry using annexin V-FITC/PI double staining kit (Cayman Chemicals, Carlsbad, USA). After the incubation period, cells were washed with cold phosphate buffered saline (PBS), centrifuged twice at 1500 rpm for 5 min, and suspended in 500 *μ*l of binding buffer. FITC-labeled annexin V (5 *μ*l) and propidium iodide (PI, 5 *μ*l) were added and incubated with the cells at room temperature for 15 min. Apoptotic cells were measured using a FACSCalibur flow cytometer (Becton Dickinson, NJ, USA). Annexin V-positive, PI-negative cells were scored as early apoptotic cells. Cells that were positive for both annexin V and PI were considered as late apoptotic cells.

### 2.4. mRNA Expression Studies by Reverse Transcription-Polymerase Chain Reaction (RT-PCR)

Total RNA was isolated from the striatal tissue using total RNA isolation reagent (TRIZOL, Invitrogen, Carlsbad, CA, USA). Oligonucleotide primer sequences (Supplementary Table 1) of the selected genes for reverse transcription-polymerase chain reaction (RT-PCR) were synthesized by Sigma-Aldrich (St. Louis, MO, USA) and Eurofins Genomics (India). The amplified products were separated by electrophoresis on 1-2% agarose gel. Specificity was confirmed by the size of the amplified products with reference to 100 bp DNA ladder (BioVision, USA) and the band intensities were quantified by Quantity One Software (Bio-Rad, USA).

### 2.5. Western Blotting

Striatal tissue lysates (50 *μ*g protein) and SH-SY5Y cell lysates were separated by SDS-PAGE on 10–12% polyacrylamide gels and transferred to polyvinylidene difluoride membranes. The membranes were incubated with specific primary antibodies and the antibodies used were DJ-1, Nrf2, phosphoS40-Nrf2 (Abcam 1 : 1000 dilution), Keap1, NQO1, Cullin3, PKC-*δ* (Pierce Antibodies, 1 : 1000 dilution), *γ*GCLC, HO-1, nitrotyrosine (Santa Cruz Biotech, 1 : 1000 dilution), *α*-synuclein, and iNOS (Cell Signaling Technology, 1 : 1000 dilution). To verify the uniformity of protein load and transfer efficiency across the test samples, membranes were reprobed with *β*-actin and Lamin B (Cell Signaling Technology, 1 : 1000 dilution). Immunoreactive bands were developed with Immobilon Western Chemiluminescent HRP substrate (Millipore Corporation, Billerica, USA) and visualized by using an enhanced chemiluminescence system (ChemiDoc, Bio-Rad, USA) and presented in comparison to *β*-actin/Lamin B expression.

### 2.6. Statistical Analysis

Data are presented as mean ± standard error of mean (SEM) of the results obtained from the average of at least three to six independent experiments. Results were analyzed by one-way analysis of variance (ANOVA) using the SPSS software package for Windows (Version 20.0; SPSS Inc., Chicago, IL, USA) and *p* values were determined using the Student-Newman-Keuls and least significant difference post hoc test. Differences among means were considered statistically significant when the *p* value was less than 0.05.

## 3. Results

### 3.1. Impact of MCE on Nigrostriatal Tyrosine Hydroxylase (TH) Immunoreactivity

**Figure 1:**
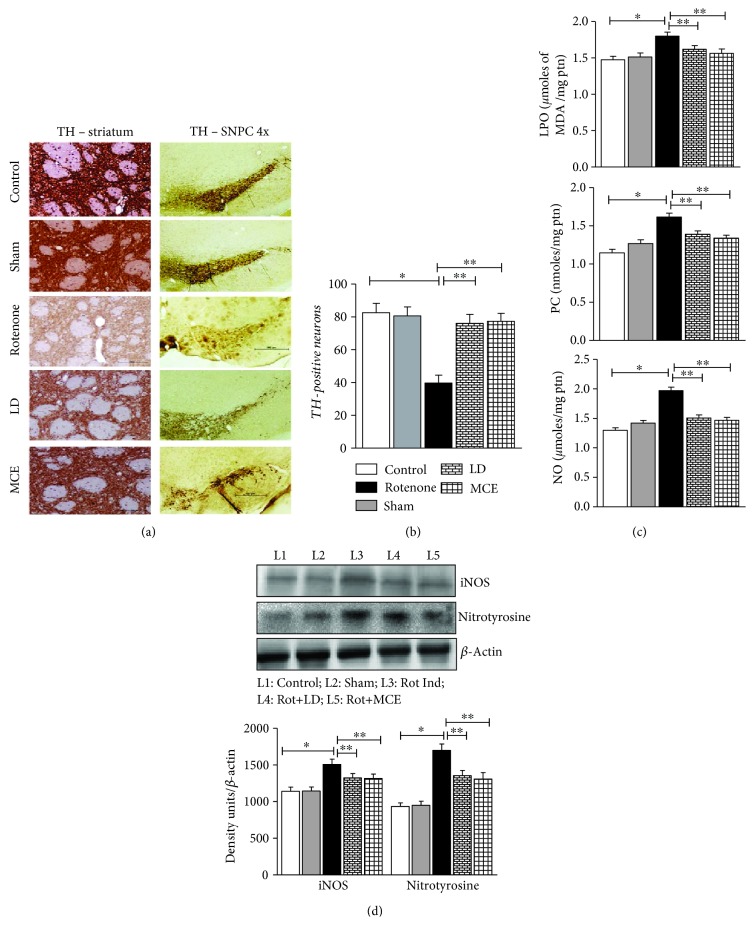
(a) Photographic representation of the TH-positive neurons in the striatum and in SNPc. (b) Quantification of relative intensity of TH staining in the striatum using the densitometry protocol through ImageJ was performed to substantiate the potentials of salvaging activity of MCE. (c) Impact of MCE on rotenone-induced oxidative stress: values are expressed as for six animals in each group. (d) Immunoblot analysis of iNOS and nitrotyrosine (i.e. α-synuclein) and representative densitometry quantification. Statistical significance (*p* < 0.05) was calculated by Student-Newman-Keuls and least significant difference post hoc test, where ^∗^ represents control vs. other groups, ^∗∗^ represents rotenone vs. LD, MCE.

Immunohistochemical localization of TH-positive neurons in the striatum as well as the substantia nigra pars compacta (SNPc) of control and experimental rats is presented in Figure 1(a). Histochemical analysis of SNPc along with the striatum makes it convenient to understand the efficacy of the MCE treatment. The striatum ipsilateral to the side of infusion showed significant loss of TH immunostaining. TH immunoreactivity of the ipsilateral striatum shows a remarkable refurbishment of dopaminergic neurons seen in MCE-administered rats. Rotenone-infused SNPc showed less number of TH-positive cells as compared to control animals. MCE treatment in these animals restored, to a great extent, the loss of these cells. While there was a 43% decrease in TH-positive neurons in response to rotenone administration, MCE treatment curtailed this to 30% (Figure 1(b)).

### 3.2. MCE Counteracts Rotenone-Induced Oxidative Stress in Experimental PD Rats

Analyses of various oxidative stress markers indicated that MCE was an efficacious treatment to reduce oxidative stress in rotenone-induced PD rats. We first determined the levels of nitric oxide (NO) which was significantly increased (*p* < 0.05) in the striatum of rotenone-infused PD rats and this was blunted in response to MCE treatment. Next, quantification of lipid peroxidation (LPO) and protein carbonyls (PC) revealed that rotenone infusion increased the levels of these oxidative by-products to 22 and 41%, respectively, in relation to sham controls (Figure 1(c)). However, upon treating with MCE, LPO and PC content was significantly decreased.

### 3.3. MCE Protects from Rotenone-Induced Protein Aggregation

Considering the interrelationships between nitric oxide, oxidative stress, and protein aggregation [14], we further determined the impact of MCE on the levels of iNOS and nitrotyrosine as the former is involved in the synthesis of NO and the latter is a marker for protein aggregation. Rotenone-infused Parkinsonian rats showed a significant (*p* < 0.05) increase in the protein levels of iNOS and nitrotyrosine when compared with controls (Figure 1(d)). Treatment with MCE significantly ameliorated rotenone-mediated NO production through diminishing the levels of iNOS. As a result, we also found that MCE was particularly efficient in blocking the formation of nitrotyrosine adducts (i.e. *α*-synuclein), which are the primary events in the process of protein aggregation that occurs in response to oxidative insults, such as those triggered by rotenone.

**Figure 2:**
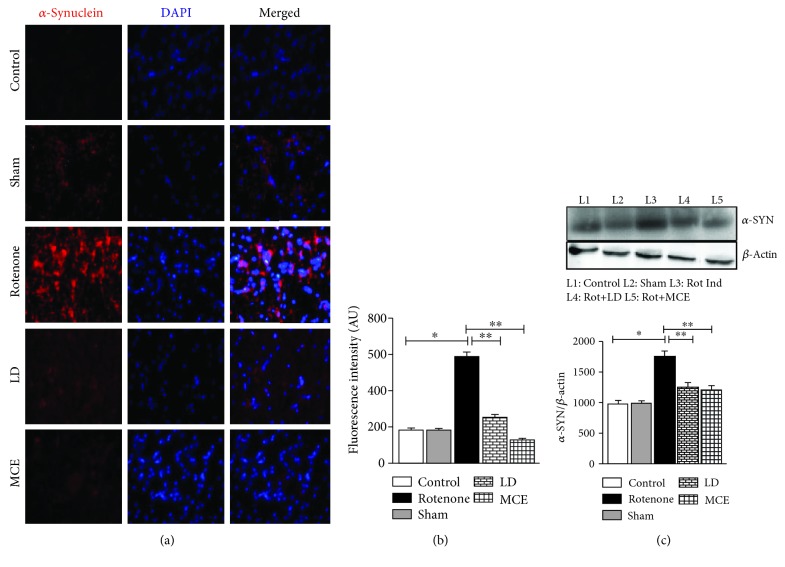
Rotenone-induced aggregation of *α*-synuclein (*α*-Syn) measured by immunofluorescence analysis. (a) The cells were visualized using fluorescence microscopy and images captured using 20x magnification. The control and sham groups show very low level of expression; however, the expression is punctate and high in the rotenone-induced group; the expression is meager in levodopa; on the contrary, expression in the MCE groups is comparable with that in the control group. (b) Relative fluorescence intensity was calculated. (c) Immunoblot analysis of *α*-Syn and representative densitometry quantification. Statistical significance (*p* < 0.05) was calculated by Student-Newman-Keuls and least significant difference post hoc test, where ^∗^ represents control vs. other groups, ^∗∗^ represents rotenone vs. LD, MCE.

To further confirm the suppression of protein aggregation by MCE, we analyzed both the protein levels and the immunostaining for *α*-synuclein (*α*-Syn), a pathological protein that is aggregated in PD. While immunostaining showed a significant increase in *α*-Syn aggregation (Figures 2(a) and 2(b)) in the striatum of rotenone-induced rats which correlates with the increase in the nitrotyrosine levels, MCE treatment abolished these changes and significantly reduced the aggregation of *α*-Syn suggesting that oxidative stress promotes the aggregation of *α*-Syn. Moreover, rotenone-infused Parkinsonian rats exhibited a significant (*p* < 0.05) increment in the protein levels of *α*-Syn by 79% (Figure 2(c)), which further aggravated the aggregation of *α*-Syn in these rats.

### 3.4. MCE Prevents Rotenone-Induced Oxidative Stress by Augmenting the Antioxidant Defense

We postulated that the antioxidative potential of MCE might be associated with diminished oxidative stress. A significant decline (*p* < 0.05) in the activities of antioxidant enzymes, namely, superoxide dismutase (SOD), catalase, glutathione peroxidase (GPx), glutathione reductase (GR), and glutathione-S-Transferase (GST), was observed (Table 1(a)) in the rotenone-induced Parkinsonian rats when compared with controls. However, upon supplementing with MCE, a significant augmentation in the activities of SOD, CAT, and GST was observed with a maximum improvement in the SOD activity (36%). However, there were no significant changes in the activities of other enzymatic antioxidants GPx, GR. In addition, the levels of reduced glutathione (GSH), a vital ubiquitous antioxidant thiol, decreased in response to rotenone-induced oxidative stress. MCE supplementation significantly increased the levels of GSH back to near normal when compared with the rotenone-infused rats. Because the Nrf2 pathway transcriptionally activates glutathione-biosynthesizing enzymes, we next assessed whether GSH changes were mechanistically linked to Nrf2 signaling in the MCE-treated animals.

### 3.5. MCE Induces Nrf2/ARE Pathway and Suppresses Rotenone-Induced Oxidative Stress

The downregulation of the Nrf2/ARE pathway exacerbates oxidative stress which potentiates dopaminergic degeneration and pathogenesis of PD [15]. As we noticed a significant alteration in cellular redox status, elevated *α*-Syn expression, and aggregation in the current study, we further analyzed the levels of Nrf2 and its interacting proteins to test the hypothesis that the MCE-mediated augmentation of antioxidative system occurs through the activation of Nrf2/ARE signaling.

**Figure 3:**
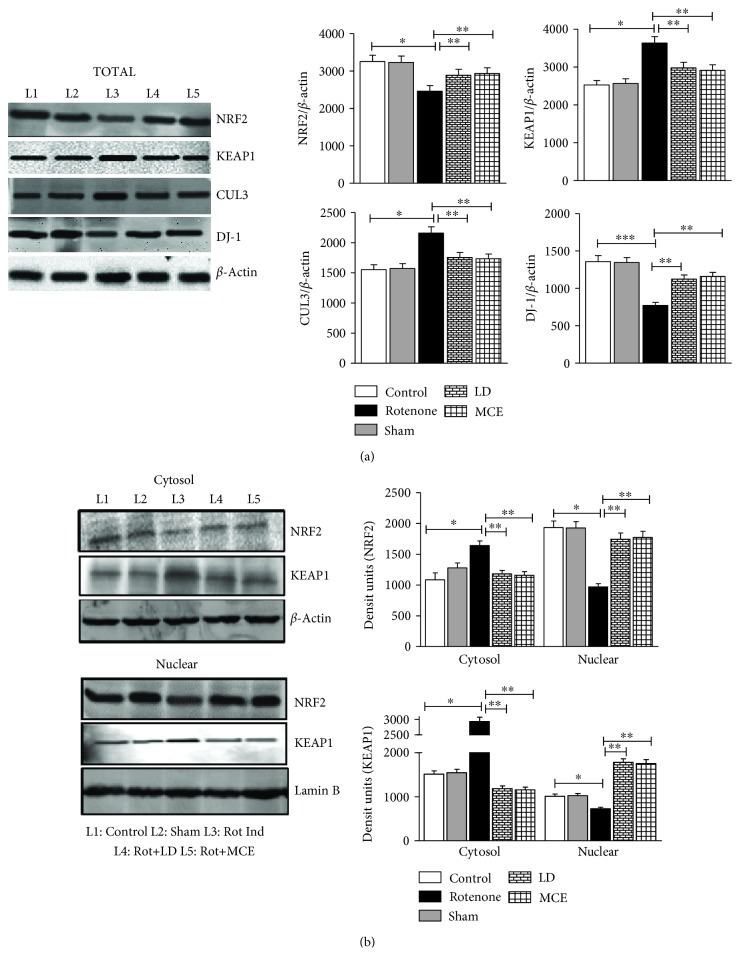
Immunoblot analysis of Nrf2 and its negative regulators Keap1 and Cullin3 in the striatum of rotenone-induced PD rats. (a) The total protein levels of Nrf2, Keap1, Cullin3, and DJ-1. (b) The protein levels of Nrf2 and Keap1 in cytosolic and nuclear compartment of striatal neuronal cells. Statistical significance (*p* < 0.05) was calculated by Student-Newman-Keuls and least significant difference post hoc test, where ^∗^ represents control vs. other groups, ^∗∗^ represents rotenone vs. LD, MCE.

Immunoblotting analysis revealed that rotenone infusion significantly decreased Nrf2 protein levels. In compounding fashion, Keap1 and Cullin3, scaffold and adaptor proteins responsible for cytosolic sequestration and proteasomal degradation of Nrf2, respectively, were significantly increased in rotenone-infused rats. Interestingly, MCE treatment significantly rescued the levels of Nrf2 and reversed the rotenone-induced increase in Keap1 and Cullin3 (Figure 3(a)). Recent reports indicate that nuclear translocation of Nrf2 is not only mediated by phosphorylation by PKC-*δ* but also by intact DJ-1, which binds and stabilizes the Nrf2 and favours its translocation to the nucleus [16]. Next, we extended our immunoblotting analyses to DJ-1; similar to that of Nrf2, the DJ-1 is also downregulated in rotenone-infused rats and this was reversed upon MCE administration (Figure 3(a)). To delineate the difference in total protein expression, we performed the immunoblotting of Nrf2 and Keap1 in nuclear and cytosolic fractions (Figure 3(b)). Our results indeed confirmed that rotenone infusion significantly repressed the translocation of Nrf2 from the cytosol to the nucleus as evident from decreased levels of nuclear Nrf2 in rotenone versus control rats. Hence, rotenone infusion not only decreases total Nrf2 protein levels but also impairs its nuclear translocation by downregulating DJ-1 and augmenting cytosolic Keap1 levels. As such, supplementation with MCE significantly augmented the nuclear translocation of Nrf2 as evident from increased levels of nuclear Nrf2 (83%) by augmenting the DJ-1 and decreasing the nuclear Keap1 levels when compared with rotenone-infused rats.

### 3.6. MCE Augments Nrf2/ARE Downstream Genes

**Figure 4:**
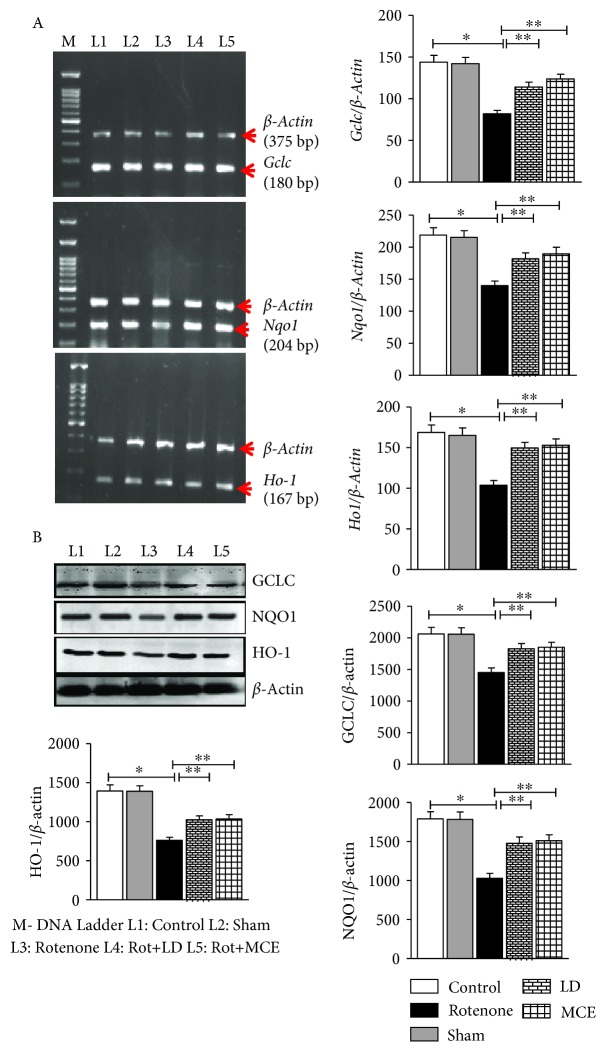
Increased Nrf2 regulated antioxidant genes in response to MCE supplementation in rotenone-induced PD rats. (a) The mRNA levels of Nrf2 downstream genes *γ*GCLC, NQO1, and HO-1. (b) The protein levels of *γ*GCLC, NQO1, and HO-1. Statistical significance (*p* < 0.05) was calculated by Student-Newman-Keuls and least significant difference post hoc test, where ^∗^ represents control vs. other groups, ^∗∗^ represents rotenone vs. LD, MCE.

Nrf2, a member of Cap'n'Collar family of basic region-leucine zipper (bZIP) transcription factors, plays an important role in ARE-mediated gene expression through the transcriptional activation of antioxidant genes such as heme oxygenase-1 (*Ho-1*), gamma-glutamyl cysteine ligase (*γGclc*), and NAD(P)H:quinone oxidoreductase 1 (*Nqo1*). As we observed a significant decline in Nrf2 protein levels, we further assayed transcript and protein levels for Nrf2 gene targets. Consistent with our observation of oxidative insult and Nrf2 pathway antagonism, rotenone treatment was associated with a prominent decline in the mRNA (Figure 4(a)) and protein (Figure 4(b)) levels of several ARE targets with a maximum decrease being observed in *Ho-1* (45%). However, supplementation with MCE significantly enhanced the levels of these proteins both at the transcriptional and translational levels by an average of 40%. The improved protein levels are reflected in the activities of these enzymes (Table 1(b)).

After characterizing the neuroprotective effect of MCE in an *in vivo* model of PD and confirming its ability to augment Nrf2 signaling and reverse oxidative insult, we further examined the neuroprotective efficacy of scopoletin, a major and active principle of MCE using SH-SY5Y cells. To choose an optimal concentration of scopoletin for this study, we pretreated SH-SY5Y neuroblastoma cells with different doses of scopoletin ranging from 1 to 100 *μ*M at different time intervals (from 0 hours to 6 hours). Until 50 *μ*M scopoletin, there was no toxicity observed in SH-SY5Y cells. Maximum viability was achieved at the concentration of 30 *μ*M scopoletin (Supplementary Figure 2). Hence, further studies were carried out using 30 *μ*M scopoletin.

### 3.7. Scopoletin Prevented Rotenone-Induced Cell Death

**Figure 5:**
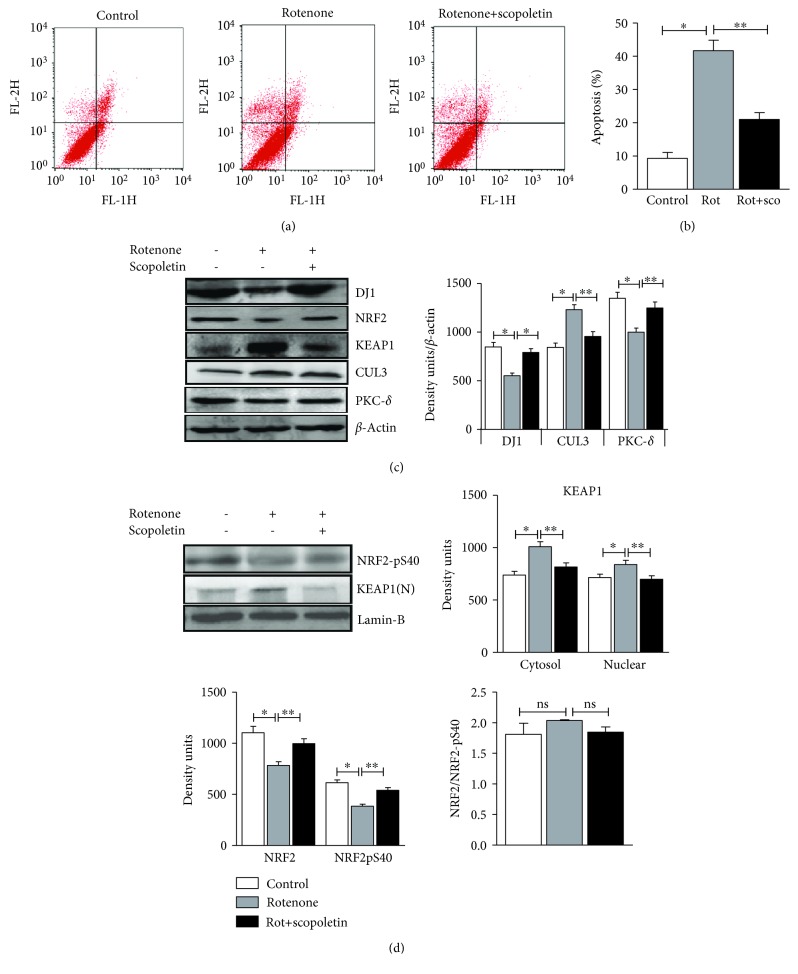
(a) Rotenone-induced apoptosis is counteracted by scopoletin in SH-SY5Y cells. At the end of the experiment, cells were stained with annexin V/FITC and read immediately by flow cytometry to measure the extent of apoptosis; *x*-axis FL-1H denotes the FITC and *y*-axis FL-2H denotes the PI. (b) Quantification of percentage dead cells. (c) Immunoblot analysis of several proteins involved in Nrf2/ARE pathway is performed in SH-SY5Y cells treated with/without rotenone and scopoletin. (d) Immunoblot analysis of phospho (S40) Nrf2 and nuclear Keap1 in SH-SY5Y cells. Statistical significance (*p* < 0.05) was calculated by Student-Newman-Keuls and least significant difference post hoc test, where ^∗^ represents control vs. other groups, ^∗∗^ represents rotenone vs. scopoletin.

SH-SY5Y cells undergoing various stages of apoptosis (early, midstage, and late stage) were analyzed by flow cytometry using annexin V and propidium iodide (PI) dual staining. Treatment of SH-SY5Y cells with rotenone (500 nM for 24 h) resulted in 40% cell death, which was attenuated by pretreating the cells with 30 *μ*M scopoletin for 3 h (Figures 5(a) and 5(b)). These data indicate that scopoletin protects SH-SY5Y from rotenone-induced cell death.

### 3.8. Scopoletin-Mediated Neuroprotective Effects Were Associated with the Translocation of Nuclear p40Nrf2 and Upregulation of DJ-1

The activation of the Nrf2/ARE pathway is known to confer resistance to oxidative stress-induced cell death [17]. As scopoletin prevented rotenone-induced cell death, we further assessed Nrf2 pathway constituents. Along these lines, both the unphosphorylated (total/cytosolic form) and the serine 40-phosphorylated Nrf2 (pNrf2S40) levels were measured by immunoblotting. Consistent with animal studies demonstrating that MCE improves nuclear levels of Nrf2, scopoletin also increased the nuclear translocation of Nrf2 as evident from the increased nuclear levels of pNrf2 (S40) (Figure 5(d)). This increase in the nuclear levels of Nrf2 may be attributed to the phosphorylation of Nrf2 by PKC-*δ* which is also augmented upon pretreating with scopoletin (Figure 5(c)). Concomitant with the animal studies, both the total and nuclear levels of Keap1 were significantly elevated in rotenone-treated SH-SY5Y cells when compared with untreated control cells. Furthermore, rotenone treatment also increased the levels of E3 ubiquitin ligase Cullin3 (Figure 5(c)) by 46%.

In order to determine whether DJ-1 plays an important role in scopoletin-mediated Nrf2 translocation, we further analyzed the levels of DJ-1 in rotenone-induced SH-SY5Y cells pretreated with or without scopoletin. Rotenone-treated cells exhibited a relative decrease in the protein levels of DJ-1 (Figure 5(c)) which may be attributed to the impaired Nrf2 nuclear translocation in these cells. Conversely, scopoletin-pretreated cells showed an augmented expression of DJ-1 (46%), suggesting that DJ-1 may be vital for scopoletin-mediated neuroprotective effects. From these observations, it is clear that scopoletin augments the Nrf2/ARE pathway by increasing levels of DJ-1 and concomitantly prevents the cytosolic degradation of Nrf2 by reducing the levels of its negative regulators Keap1 and Cullin3.

## 4. Discussion

Increasing interest has been focused on identifying dietary supplements and phytoconstituents that can inhibit ROS-mediated protein aggregation and neuronal cell death and thereby reverse the multifaceted pathophysiological events underlying PD. Here, we investigated whether *Morinda citrifolia* fruit extract attenuates rotenone-induced oxidative stress by activating the Nrf2-dependent antioxidant response and tested whether this mechanism may prevent the loss of dopaminergic neurons. After confirming its neuroprotective effect *in vivo*, we elucidated the mechanism of action for scopoletin, a major compound present in the MCE, *in vitro* using SH-SY5Y cells and identified that the therapeutic effect of scopoletin is facilitated through the activation of the DJ-1/Nrf2/ARE signaling cascade.

While the pathogenic mechanism of PD is poorly known, it is believed that oxidative stress involving the imbalance of nitric oxide (NO) signaling is a major player in the prognosis of PD [14]. A significant increase in the levels of NO in the striatum of rotenone-infused Parkinsonian rats is in line with previous reports [18]. However, supplementation of MCE reduced the levels of NO in the striatum of PD-induced rats. Recent reports documented that the ethyl acetate extract of noni fruit is shown to reduce oxidative and nitrosative stress in the brain [19]. As NO homeostasis is significantly impaired in response to rotenone infusion, our further analysis revealed an increase in the levels of iNOS in the striatal tissues in these rats. Thus, the increased iNOS might have been responsible for increased nitric oxide levels. The administration of MCE safeguarded the striatum from the deleterious effect of nitric oxide by attenuating the iNOS expression induced by rotenone (Figure 1).

As protein aggregation is a common event underlying neurodegenerative diseases including PD, wherein *α*-synuclein (*α*-Syn) comprises the bulk of Lewy bodies [20, 21], further investigations were directed to assess the rate of *α*-Syn aggregation. *α*-Syn expression and its aggregation were increased in the striatum of rotenone-infused rats when compared with the control rats (Figure 2). This increased aggregation of *α*-Syn might also be due to NO-mediated oxidative stress in PD rats (Table 1(a)), as it has been previously shown that fibrillary *α*-Syn aggregates with perinuclear localization were formed in cells exposed to NO [22]. Interestingly, treatment with MCE reduced the *α*-Syn aggregation which might be due to the reduction in the levels of NO and the consequent tyrosine nitration in MCE-treated rats.

Intracellular defense is maintained through a variety of antioxidant enzymes and low molecular weight antioxidants such as glutathione to combat the deleterious effects of ROS overproduction and oxidative damage [23]. Elevated ROS production, in the absence of increased antioxidant defenses, will exacerbate oxidative damage and oxidative stress [24]. In the current study, an overall decline in the activities of antioxidant enzymes such as superoxide dismutase (SOD), catalase (CAT), glutathione peroxidase (GPx), glutathione reductase (GR), and glutathione-s-transferase (GST) was noticed along with significantly decreased glutathione (GSH) content in the striatum of intranigrally rotenone-infused rats when compared with control rats. Notably, MCE supplementation restored the overall antioxidant status, rescuing glutathione levels in the striatum of rotenone-infused PD rats. These results support recent work demonstrating that the ethyl acetate extract of *Morinda citrifolia* fruit boosts SOD, GPx, and GR enzymatic activity in *β*-amyloid-induced cognitive dysfunction in mice [25] and in the skeletal muscle of rotenone-infused hemi-Parkinsonian rats [10].

In the present investigation, the total protein level of Nrf2 was significantly reduced in rotenone-induced rats while Keap1 and Cullin3, negative regulators of Nrf2, were markedly elevated. High levels of oxidative stress may reduce the activity of Nrf2, although the molecular mechanism for this defect is uncertain [26]. Interestingly, the administration of MCE reversed the rotenone-mediated increase in Keap1 and Cullin3, thereby preventing the degradation of Nrf2 *in vivo*. Keap1 is capable of restraining Nrf2 activity not only *via* its capacity to target Nrf2 to a cytoplasmic Cullin3-based E3 ligase [27] but also by transiently entering into the nucleus and targeting Nrf2 for ubiquitylation in this compartment under stressed conditions [28]. This instigated us to analyze the nuclear levels of Nrf2 and Keap1 which gives us an unblemished picture of the nuclear translocation and activated form of Nrf2.

The nuclear level of Nrf2 was reduced in the striatum of rotenone-infused Parkinsonian rats when compared with the control rats and the opposite trend was observed for Keap1. From these observations, it may be inferred that not only is a lower concentration of Nrf2 protein present in the nucleus but that it also may be bound by Keap1, thereby preventing Nrf2 from binding to AREs. Indeed, this notion is consistent with our observation of a lower antioxidant status in these rats. However, the redox milieu inside the striatal nucleus of PD rats treated with MCE is different, where MCE with its rich phytoconstituents favours the translocation of Nrf2 into the nucleus and detains Keap1 in the cytosol, which is further reflected in the results obtained for antioxidant status in these rats. From these observations, it is clear that MCE increases the nuclear translocation of Nrf2.

Since we observed a significant decline in the transcriptional activity of Nrf2, we further assessed its downstream effectors (*γGclc*, *Nqo1* and *Ho-1*) by analyzing the levels of mRNA and protein. Overall, both the mRNA and protein levels of *γGclc*, *Nqo1* and *Ho-1* were significantly decreased in the striatum of rats stereotaxically infused with rotenone when compared with the control rats. The consequence of impaired transcription and translation of these proteins was also reflected in the diminished activities of these enzymes on rotenone infusion. However, upon treating with MCE, the activities and mRNA and protein levels of these proteins were significantly increased, likely a downstream effect of enhanced Nrf2 stability and activation.

Overall, from the *in vivo* studies, it is clear that MCE, with its antioxidant property, scavenges the free radicals, and it also reduces the expression of iNOS and prevents rotenone-induced aggregation of *α*-synuclein. MCE also augments the total levels of Nrf2 and subsequently translocates Nrf2 to the nucleus by preventing its degradation mediated by Keap1/Cullin3 complex which in turn leads to transcription and enhanced activities of its downstream effectors *γGclc*, *Ho-1* and *Nqo1*. This neuroprotective effect of MCE might be attributed to the presence of identified phytoconstituents quercetin, rutin, and scopoletin and also other unidentified constituents [10]. However, as scopoletin is the biomarker for *Morinda citrifolia* [29] while other compounds such as rutin and quercetin are commonly present in most of the plants [30, 31], further emphasis was given to scopoletin, and its mechanism of action in boosting DJ-1/Nrf2/ARE pathway was studied in *in vitro* cell culture using SH-SY5Y dopaminergic cells, in order to delineate the ability of the phytochemical derived from *Morinda citrifolia* on dopaminergic neuronal cell survival.

We observed a significant reduction in cell viability on rotenone-exposed SH-SY5Y cells (data not shown), which was attenuated by pretreatment with scopoletin. Hence, we further analyzed the status of cellular apoptosis using annexin V/propidium iodide staining. Rotenone exposure of SH-SY5Y cells shows a significant increase in the proportion of early apoptotic cells compared with the controls. Our observation is in coherence with the previous reports by Jang et al. [32], who have stated that rotenone at a concentration of 200 nM induces apoptosis in SH-SY5Y cells by generating ROS. However, this shift is reversed when the cells are pretreated with scopoletin, and the possible explanation for this effect would be scopoletin with its antioxidant potential [33] should have ameliorated rotenone-induced apoptosis by quenching free radicals. To the best of our knowledge, this is the first study to show that scopoletin prevents apoptosis in an *in vitro* rotenone exposure unless otherwise like literature which poses it as a potent proapoptotic agent in various cancer cell lines [34–36].

In light of our observations on MCE-mediated Nrf2 activation, we examined the capacity of scopoletin to influence the Nrf2 signaling by aiding the nuclear translocation of Nrf2. We analyzed the levels of phospho-Nrf2 and PKC-*δ* that phosphorylate serine 40 of Nrf2, thereby aiding in its nuclear translocation, in SH-SY5Y cells exposed to rotenone. Pretreatment with scopoletin augmented the nuclear levels of phospho-Nrf2 which may be associated with our observation of increased PKC-*δ* expression. Indeed, Nam and Kim [37] have shown that scopoletin influences the expression of reprogramming genes and exerts antiaging effects by regulating the transcription factor Nrf2 (Figure 5). Previous reports have shown that DJ-1 stabilizes Nrf2 and promotes its nuclear translocation [38]. Intriguingly, mutations in DJ-1 are associated with the risk of developing PD [39]. Hence, we further evaluated the effect of scopoletin on DJ-1 levels in SH-SY5Y cells. We found that rotenone exposure of cells resulted in diminished levels of DJ-1. Angeline et al. [40] have reported that chronic exposure to rotenone reduced the cytoprotective proteins Parkin, Hsp70, and DJ-1. However, on pretreating with scopoletin, DJ-1 protein levels were augmented, subsequently conferring protection against rotenone-induced oxidative stress, as overexpression of DJ-1 rescued MN9D cells exposed to rotenone, indicating that DJ-1 protected nigral DA neurons from rotenone-induced cell death [41].

## 5. Conclusions

This study has provided evidence that MCE prevented *α*-synuclein aggregation through augmenting Nrf2 antioxidant signaling, leading to the suppression of oxidative stress. Notably, scopoletin, the active component from MCE, seems to be responsible for stabilizing Nrf2/ARE pathway by augmenting the phosphorylation of Nrf2 and its nuclear translocation, in a DJ-1-dependent manner. Thus, we propose that DJ-1 might be a potential target for scopoletin-based therapeutic strategy against neurodegenerative diseases.

## Figures and Tables

**Table 1a:** (a) Impact of MCE on antioxidant defense system

Parameter	Control	Sham control	Rotenone induced	LD	MCE
SOD	0.58 ± 0.018	0.52 ± 0.017	0.33 ± 0.015^∗^	0.41±0.016^∗∗^	0.45±0.014^∗∗^
CAD	17.43 ± 0.60	16.36 ± 0.36	12.13 ± 0.38^∗^	14.21±0.32^∗∗^	15.22±0.41^∗∗^
GPx	8.25 ± 0.37	8.22 ± 0.32	5.66 ± 0.23^∗^	6.59±0.31^∗∗^	6.64±0.20^∗∗^
GR	5.16 ± 0.25	5.12 ± 0.22	3.90 ± 0.15^∗^	4.47±0.19^∗∗^	4.48±0.15^∗∗^
GST	9.08 ± 0.394	9.01 ± 0.468	6.38 ± 0.271^∗^	7.75±0.298^∗∗^	7.86±0.241^∗∗^
GSH	1.93 ± 0.055	1.76 ± 0.048	1.28 ± 0.058^∗^	1.61±0.059^∗∗^	1.65±0.062^∗∗^

**Table 1b:** (b) Impact of MCE on the activities of Nrf2/ARE downstream enzymes

Parameter	Control	Sham control	Rotenone induced	LD	MCE
*γ*GCL	2.54 ± 0.084	2.46 ± 0.083	1.35 ± 0.051^∗^	1.90±0.071^∗∗^	2.01±0.068^∗∗^
NQO1	5.88 ± 0.193	5.85 ± 0.193	3.15 ± 0.111^∗^	4.57±0.151^∗∗^	4.82±0.153^∗∗^
HO-1	0.81 ± 0.026	0.80 ± 0.026	0.48 ± 0.016^∗^	0.63±0.021^∗∗^	0.65±0.018^∗∗^

## Data Availability

The data supporting the findings of this study are available within the article [and/or] its supplementary materials.

## Abbreviations

DMSO: Dimethyl sulfoxide
F-12K: Kaighn's modification of Ham's F-12 medium
FBS: Fetal bovine serum
GSH: Reduced glutathione
HO-1: Heme oxygenase-1
Keap1: Kelch-like erythroid cell-derived protein with CNC (Cap “n” Collar) homology (ECH) protein1
LD: Levodopa
LPO: Lipid peroxidation
MCE: Ethyl acetate extract of *Morinda citrifolia* fruit
NO: Nitric oxide
NQO1: NAD(P)H:quinone oxidoreductase 1
Nrf2: Nuclear factor erythroid 2-related factor 2
PC: Protein carbonyl
PD: Parkinson's disease
PKC-*δ*: Protein kinase C delta
ROS: Reactive oxygen species
Rot Ind: Rotenone induced
SH-SY5Y: Neuroblast-like subclone of SK-N-SH
SNPc: Substantia nigra pars compacta
VTA: Ventral tegmental area
*γ*GCLC: Catalytic subunit of gamma glutamate cysteine ligase.

## Supplementary Materials

Supplementary Materials The supplementary materials (SM) provided along with this manuscript details about the methods we used to determine the levels of oxidative stress markers, activities of antioxidant defense systems/downstream enzymes of Nrf2/ARE signaling pathway, immunofluorescence of *α*-synuclein, and sequence of the primers used in the PCR protocol. In addition, the SM also provides a data for behavioural analysis (Supplementary Figure 1) and dose determination of scopoletin using MTT assay (Supplementary Figure 2).
