# Supplementary material for: Morinda citrifolia and Its Active Principle Scopoletin Mitigate Protein Aggregation and Neuronal Apoptosis through Augmenting the DJ-1/Nrf2/ARE Signaling Pathway
Source: Oxid Med Cell Longev. 2019 May 2;2019:2761041. doi: 10.1155/2019/2761041 (PMC6525839; doi:10.1155/2019/2761041)
Supplement: Supplementary Materials — The supplementary materials (SM) provided along with this manuscript details about the methods we used to determine the levels of oxidative stress markers, activities of antioxidant defense systems/downstream enzymes of Nrf2/ARE signaling pathway, immunofluorescence of α-synuclein, and sequence of the primers used in the PCR protocol. In addition, the SM also provides a data for behavioural analysis (Supplementary Figure 1) and dose determination of scopoletin using MTT assay (Supplementary Figure 2). [file 2761041.f1.pdf]

Methods

Oxidative Stress Markers

The levels of nitric oxide [1] and protein carbonyls [2] and lipid peroxide [3] were assayed as described previously.

Assessment of Antioxidant Defence Systems

The Superoxide dismutase (SOD) enzyme was assayed according to the method of Marklund and Marklund [4]. The activity of Catalase (CAT) was assayed by the method of Sinha [5]. The activity of Glutathione Peroxidase (GPx) was determined by the modified method of Rotruck [6]. Glutathione Reductase (GR) which utilizes NADPH to convert oxidized glutathione to the reduced glutathione was assayed by the method of Staal [7]. The level of reduced glutathione (GSH) was determined by the method of Moron [8].

Determination of the activities of Nrf2 down-stream enzymes

Glutamyl Cysteinyl Ligase (GCLC) was assayed by the method of Fraser [9]. NAD(P)H:Quinone Oxidoreductase1 (NQO1) activity was measured by the method of Merker [10]. The enzyme activity of Heme oxygenase-1 (HO-1) was assayed by the method of Balla [11].

Methods

Immunohistochemistry and immunofluorescence

For immunohistochemical studies, the animals were anaesthetized with Ketamine (22mg i/p), perfused intracardially initially with 0.1 M phosphate-buffered saline (PBS) and followed by 4% para-formaldehyde (PFA) in 0.1 M PBS, pH 7.4. Brains were removed and fixed in 4% PFA and allowed to impregnate in 30% sucrose solution in PBS. Coronal sections passing through striatum and SNpc were cut on a microtome (7µm thickness for IHC) and cryostat (20µm thickness for immunofluorescence), collected on to gelatin subbed slides using PBS.

The sections were incubated with the primary antibody (rabbit monoclonal tyrosine hydroxylase primary antibody, 1:1,000, Pierce antibodies) in TBS, pH 7.4, containing 2% NGS and 0.2% Triton X-100 for 24 h at 4°C on a platform shaker. After rinsing in TBS, sections were incubated with secondary anti-rabbit IgG-conjugated horseradish peroxidase antibody (1:1000) in TBS, pH 7.4, containing 2% NGS for 45 min at room temperature. Visualization was performed by incubation in 3, 3-diaminobenzidine for 5 min after which the sections were examined under the light microscope (Nikon Eclipse Ti series). The average optical density of the immunohistochemical staining for TH in striatal region per unit area from sections each at comparable levels from animals was done in a blinded fashion by different observers using densitometry protocol through Image-J was performed to determine the staining intensity.

For immunofluorescence, the slides were then brought to room temperature and baked briefly in a slide drier to prevent the sections from floating away. The sections were washed thrice with PBS to ensure that the traces of cryo embedding media are removed. The sections were then blocked with 1 % Bovine serum albumin for an hour and then incubated with primary antibody (α-synuclein; Cell Signaling Technology, diluted at 1: 200) overnight at 4°C in a humid chamber. The sections were washed with PBS, after washing the sections were incubated with secondary antibodies conjugated with rhodamine for 1 hour in 37°C. The sections were then washed and stained with DAPI and cover slipped with glycerol mounting. These sections were scanned and analysed immediately for immunofluorescence signal using Nikon Eclipse Ti series and densitometry protocol through Image-J was performed as mentioned earlier.

Methods

Supplementary Table 1 PCR Primer sequences and conditions used

S.No	Gene Name	Primer sequence (5'- 3')		Annealing temperature ° C	Amplicon size
1	*Gclc*	F	TGATTGAAGGGACACCTGGC	60	180 bp
		R	TGTGCTCTGGCAGTGTGAAT		
2	*Ho-1*	F	GAGCGAAACAAGCAGAACCC	59	167 bp
		R	ACCTCGTGGAGACGCTTTAC		
3	*Nqo1*	F	CGAAGCATTTCAGGGTCGTC	60	204 bp
		R	AGATTCGACCACCTCCCATC		
4	*β-actin*	F	GCCATGTACGTAGCCATC	59	375 bp
		R	GAACCGCTCATTGCCGAT		

Supplementary Figure 1 Effect of MCE/LD in the motor function (rotarod, catalepsy and open field) of rotenone infused Parkinsonian rats

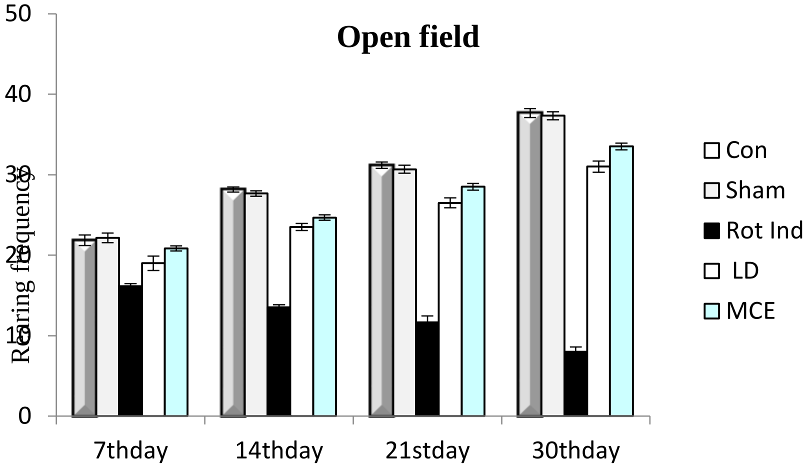

Values are expressed as mean ± SEM for six experiments in each group. Values are statistically significant at the level of $p < 0.05$ where ‘a’ represents Control Vs other groups, ‘c” represents Rot Ind vs LD, MCE.

Supplementary Figure 2 Dose determination of scopoletin using MTT assay

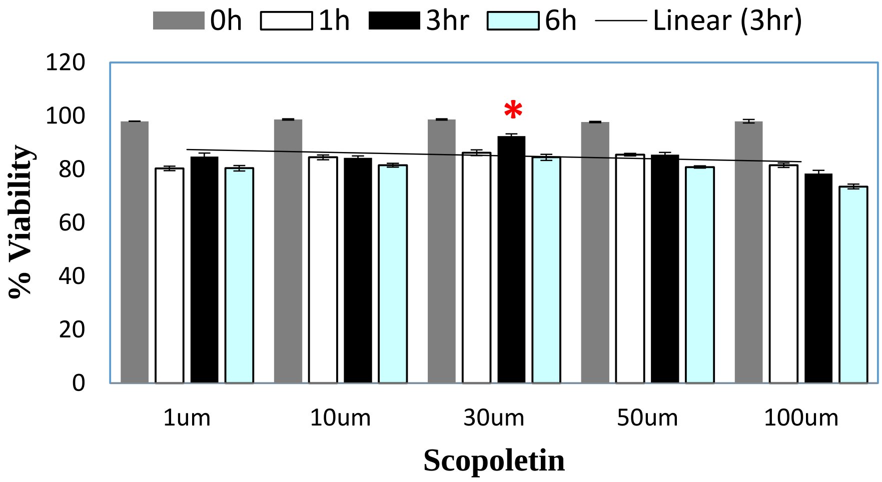

Figure 2 - A bar graph showing the cell viability determined by MTT assay. Pre-treatment with scopoletin (1, 10, 30, 50 and 100 µM at different time intervals 0 hours to 6 hours) to the culture with rotenone (500 nM) reduced rotenone-induced cell death. * The maximum protection was achieved at the concentration of 30µM scopoletin pre-treated 3 hours before rotenone treatment. Hence further studies were carried out using 30µM scopoletin.

References

[1] L.C. Green, D.A. Wagner, J. Glogowski, P.L. Skipper, J.S. Wishnok, S.R. Tannenbaum, Analysis of nitrate, nitrite, and [15 N] nitrate in biological fluids, Anal. Biochem. 126 (1982) 131–138. doi:10.1016/0003-2697(82)90118-X.

[2] R.L. Levine, D. Garland, C.N. Oliver, A. Amici, I. Climent, A.-G. Lenz, B.-W. Ahn, S. Shaltiel, E.R. Stadtman, [49] Determination of carbonyl content in oxidatively modified proteins, in: Oxyg. Radicals Biol. Syst. Part B Oxyg. Radicals Antioxidants, Academic Press, 1990: pp. 464–478. doi:https://doi.org/10.1016/0076-6879(90)86141-H.

[3] T.P.A. Devasagayam, C.K. Pushpendran, J. Eapen, Differences in lipid peroxidation of rat liver rough and smooth microsomes, Biochim. Biophys. Acta - Lipids Lipid Metab. 750 (1983) 91–97. doi:https://doi.org/10.1016/0005-2760(83)90207-2.

[4] S. MARKLUND, G. MARKLUND, Involvement of the Superoxide Anion Radical in the Autoxidation of Pyrogallol and a Convenient Assay for Superoxide Dismutase, Eur. J. Biochem. 47 (1974) 469–474. doi:10.1111/j.1432-1033.1974.tb03714.x.

[5] A.K. Sinha, Colorimetric assay of catalase, Anal. Biochem. 47 (1972) 389–394. doi:https://doi.org/10.1016/0003-2697(72)90132-7.

[6] A.J.T. Rotruck, A.L. Pope, H.E. Ganther, A.B. Swanson, D.G. Hafeman, G. Hoekstra, B.J. Tioffer, F.E. Bloom, A.L. Steiner, Selenium : Biochemical Role as a Component of Glutathione Peroxidase Published by : American Association for the Advancement of Science Stable URL : http://www.jstor.org/stable/1735448 REFERENCES Linked references are available on JSTOR for this article :, 179 (2016) 588–590.

[7] G.E.J. Staal, P.W. Helleman, J. De Wael, C. Veeger, Purification and properties of an abrnomal glutathione reductace from human erythrocytes, Biochim. Biophys. Acta - Enzymol. 185 (1969) 63–69. doi:https://doi.org/10.1016/0005-2744(69)90282-4.

[8] M.S. Moron, J.W. Depierre, B. Mannervik, Levels of glutathione, glutathione reductase and glutathione S-transferase activities in rat lung and liver, Biochim. Biophys. Acta - Gen. Subj. 582 (1979) 67–78. doi:https://doi.org/10.1016/0304-4165(79)90289-7.

[9] J.A. Fraser, P. Kansagra, C. Kotecki, R.D.C. Saunders, L.I. McLellan, The Modifier Subunit of Drosophila Glutamate-Cysteine Ligase Regulates Catalytic Activity by Covalent and Noncovalent Interactions and Influences Glutathione Homeostasis in Vivo, J. Biol. Chem. 278 (2003) 46369–46377. doi:10.1074/jbc.M308035200.

[10] M.P. Merker, R.D. Bongard, N.J. Kettenhofen, Y. Okamoto, C. a Dawson, Intracellular redox status affects transplasma membrane electron transport in pulmonary arterial endothelial cells., Am. J. Physiol. Lung Cell. Mol. Physiol. 282 (2002) L36-43. doi:10.1152/ajplung.00283.2001.

[11] G. Balla, H.S. Jacob, J. Balla, M. Rosenberg, K. Nath, F. Apple, J.W. Eaton, G.M. Vercellotti, Ferritin: A cytoprotective antioxidant strategem of endothelium, J. Biol. Chem. 267 (1992) 18148–18153.
